# Postpartum Depression among Mothers in a Maternity Hospital Kathmandu, Nepal: A Mixed Method Approach

**DOI:** 10.31729/jnma.8746

**Published:** 2024-09-30

**Authors:** Maryada Neupane, Manita Bartaula, Simran Pradhan, Hom Prasad Adhikari, Lalita Shrestha, Puja Sharma, Nishchal Devkota

**Affiliations:** 1 Nobel College, Sinamangal, Kathmandu, Nepal; 2 National Open College, Sanepa, Lalitpur, Nepal; 3 Suvekchya International Hospital, Kathmandu, Nepal; 4 Paropakar Maternity and Women's Hospital, Thapathali, Kathmandu, Nepal

**Keywords:** *maternity hospital*, *mental health*, *postpartum depression*, *women*

## Abstract

**Introduction:**

Postpartum depression, a significant global concern yet a neglected domain that could have dire outcomes for both mother and children. This study aimed to find out the prevalence of postpartum depression among mothers in the Paropakar Maternity and Women's Hospital & explore the experiences of undergoing pregnancy and post-partum melancholy.

**Methods:**

A cross-sectional descriptive study with a mixed method approach and consecutive enumeration of sampling units were done among participants at a maternity hospital, within the study period from July to December, 2023 after obtaining ethical approval (Reg no: 63/1992). Data collection was done through in-depth interviews for the qualitative part among the six respondents and self-administrative questionnaires were opted for the quantitative part among all the respondents.

**Results:**

Among the 271 respondents, 203 (74.87%) fall within the 20-30 age range. Among them, 33 (12.24%) mothers experienced post-partum depression. Pregnancy and postpartum experiences were complex, with physical and mental challenges. Women often endured painful pregnancies, including excessive vomiting and pain. Mothers lacked support from family and spouses, impeding daily life and newborn care. Traditional superstitions often trumped medical advice, leading to confusion and risks. Despite challenges, some developed coping mechanisms, like self-counselling and community support.

**Conclusions:**

The study highlights the importance of pre-pregnancy assessment and depression screening for postpartum care. Social support is crucial, necessitating holistic support with education, mental health services, and destigmatization efforts for postpartum struggles.

## INTRODUCTION

Postpartum depression (PPD) is a major depressive disorder starting 2-4 weeks after childbirth, affecting maternal and infant well-being, with symptoms ranging from mood swings to severe cases like psychosis if untreated.^12^

Postpartum depression prevalence varies widely, from 6.9% to 12.9% in high-income countries and over 20% in some low- and middle-income countries.^3^ A 2021 meta-analysis reported postpartum depression prevalence rates of 22.32% in Southern Asia, 13.53% in South-Eastern Asia^4^ and 14% to 33% in Nepal.^5,6^ Studies across Nepal have identified postpartum depression as a major public health concern.^7-9^

Gaps in evidence for postpartum depression (PPD) include limited research on effective screening methods and a lack of understanding of cultural perceptions and women's concerns, highlighting need for further study in these areas.^10^ This study aimed to assess the prevalence of postpartum depression among mothers at Paropakar Maternity and Women's Hospital and explore their experiences of pregnancy and postpartum conditions.

## METHODS

The study, conducted at Paropakar Maternity and Women's Hospital in Kathmandu from July 2023 to December 2023, utilized a mixed-method approach, including quantitative and qualitative methods. Ethical clearance was obtained (reg no: 63/1992), and data were collected from postpartum mothers who delivered within ten weeks preceding the study. Only six out of 33 possibly depressive participants consented to in-depth interviews.

The required sample size for this study was 271 which was calculated using the formula:^11^


$$\begin{array}{ll} \text{n} & ={\text{Z}}^{2}\times \frac{\text{p}\times \text{q}}{{\text{e}}^{\text{2}}}\\ & =1.96^{2}\times \frac{0.229\times 0.771}{0.05^{2}}\\ & =271 \end{array}$$

Here,

n = minimum required sample size
z = standard normal variate and equals 1.96 at a 95% Confidence Interval (CI)
p = p is prevalence of post-partum depression (22.9%) taken from a reference.^12^
q = 1-p, = 0.771
e = margin of error, 5%

We took a sample size of 271 in our analysis. The study used a self-administered questionnaire to gather information on socio-demographic, cultural, obstetric, and service utilization related information, with the first author offering guided assistance in completing the questionnaires. The Edinburgh Postnatal Depression Scale assessed depression severity. The scale has a validity of 0.86-0.92 and reliability of 0.78-0.96.^13^ The data were verified from the standard maternal card of the participants to reduce responder bias.^14^ Mostly, mothers were met at post-natal wards while some mothers were interviewed at Neonatal Intensive Care Unit (NICU) & Post Natal out patients department (OPD). For the qualitative component, in-depth interviews were conducted by first author using unstructured questions to explore postpartum experiences and coping strategies among six mothers potentially experiencing depression. The interview guideline was developed by first & last authors and reviewed by all the remaining authors. All the feedbacks were considered and guideline was amended by last author. The initial analysis to categorize depression was completed prior to these interviews. Questions were focused on the experiences of pregnancy and postpartum status, related taboos and coping techniques to deal with the challenges of the pregnancy and post-partum situation. All six in-depth interviews were taken through telephonic conversation by first author since the respondents preferred to do so. These six were taken in the qualitative component since they were approachable and were willing for the consent among all the PPD mothers. The interviews were taken in Nepali language, recorded, transferred in the hard drive & deleted from the phone recorder later. Quantitative data were entered into KOBO^15^, linked with Excel, then exported to SPSS for analysis. For qualitative analysis, recorded interviews were decoded, transcribed verbatim, and anonymized for confidentiality. Authors collectively developed codes. First and last authors conducted final coding ultimately aligning the verbatims to the themes, resolving differences together. Initial stage involved manual code development and data summarization by first, second, third and last authors however peer debriefing was conducted among the authors to ensure the accuracy of the translation of verbatim transcripts. An abductive approach generated codes from predetermined topics and aligned with overarching themes. All authors collaboratively reviewed and reached consensus on the generated themes.

## RESULTS

A total of 271 patients were surveyed, with 203 (74.87%) patients were aged 20-30 and 12 (4.43%) were younger than 20. There were 202 (74.54%) Hindu participants, with 65 (23.98%) being Buddhist, and other religions represented. The residence of 109 (40.20%) participants was in a municipality, 96 (35.49%) participants lived in a metropolitan, and 28 (10.31%) in a sub-metropolitan city. (Table 1).

**Table 1:** Socio-demographic characteristics (n=271).

Characteristics	n (%)
**Age Group**	
<20	12 (4.43)
20-30	203 (74.91)
>30	56 (20.66)
**Ethnicity**	
Dalit	32 (11.81)
Janajati	146 (53.87)
Madhesi	15 (5.54)
Brahmin/Chhetri	77 (28.41)
Muslim	1 (0.37)
**Religion**	
Hindu	202 (74.54)
Buddhist	65 (23.98)
Islam	1 (0.37)
Christian	2 (0.74)
Others	1 (0.37)
**Family Type**	
Nuclear	211 (77.86)
Joint	12 (4.43)
Extended	48 (17.71)
**Education Status**	
Only able to read and write	31 (11.44)
Primary education	94 (34.69)
Secondary education	97 (35.79)
Bachelors, Masters or above	49 (18.08)
**Residency**	
Municipality	109 (40.20)
Rural municipality	38 (14.00)
Sub-metropolitan city	28 (10.31)
Metropolitan	96 (35.49)

Among the respondents 268(98.89%) reported no intimate partner violence. Oul of the respondents, 194 (71.48%) had no gender preference for their child; pregnancy was planned in 216 (79.67%) respondents. Among the respondents, postpartum depression was present in 33 (12.24%) of them (Table 2).

**Table 2:** Cultural, obstetric and health service utilization related characteristics (n = 271).

Characteristics	n (%)
**Intimate partner violence**	
No violence	268 (98.89)
Psychological	1 (0.37)
Sexual	1 (0.37)
Physical	1 (0.37)
**Gender preference**	
Son	37 (13.71)
Daughter	40 (14.81)
Either one of them	194 (71.48)
**Mode of Pregnancy**	
Planned	216 (79.67)
Unplanned	55 (20.33)
**Mode of Delivery**	
Vaginal delivery	117 (43.22)
Caesarean birth	154 (56.78)
**Vaginal tear**	
No	249 (91.88)
Yes	22 (8.12)
**Gestational Diabetes**	
No	260 (95.92)
Yes	11 (4.08)
**Pregnancy Induced High Blood Pressure**	
No	260 (95.92)
Yes	11 (4.08)
**Antenatal Checkup**	
No	9 (3.33)
Yes (completed one)	262 (96.67)
**Intra natal Checkup**	
No	3 (1.12)
Yes	268 (98.88)
**Postnatal Checkup**	
No	5 (1.85)
Yes (completed one)	266 (98.15)
**Doctor's behaviour towards patient**	
Unsatisfied	3 (1.12)
Neutral / Undecided	67 (24.81)
Satisfied	201 (74.07)
**Nurse's behaviour towards patient**	
Unsatisfied	5 (1.85)
Neutral / Undecided	72 (26.67)
Satisfied	194 (71.48)
**Postpartum Depression**	
No	238 (87.76)
Yes	33 (12.24)

**Qualitative findings**

**Pregnancy and postpartum related experiences**

During pregnancy, numerous women experience distressing symptoms like severe vomiting and discomfort, yet societal emphasis on physical health often eclipses the importance of mental wellbeing, leading to insufficient support and awareness regarding mental health needs. The findings are supported by the responses from the participants as:

*’I had a very bad experience during this pregnancy. Maybe the reasons were my increasing age and being near menopause along with sudden body change. It was a near death experience for me and I thought I would die.' -Female, 40s, urban.*

*'I used to overthink about the upbringing of the child, doubt whether to have a child or not, be triggered by small things, and cry over small things. However, things went well further, the labour pain. How can I share those feelings with you?' -Female, 30s, urban*

*'It was a difficult pregnancy for me this time compared to the previous ones. I had excessive nausea and vomiting, abdominal pain as well as loss of appetite in this time. All these were new to me and I was worried if something might have been wrong'-Female, 20s, rural*

New mothers often struggle with inadequate support from family and spouses, limited traditional care advice, and difficulties expressing their feelings. Society prioritizes newborns over mothers' mental and physical needs, leaving them confused and unprepared for postpartum challenges.

*'Giving birth to a child is easier than dealing with postpartum care. The numerous sleepless nights lead to irritation and a sense of heaviness. Being alone, particularly for me, made it difficult to cope with daily tasks without any family support. Additionally, due to stitches, I had to take a whole month's bed rest, which has created even a greater challenge for me.' -Female, 20s, urban*

*'It is quite a difficult situation as I had a C-section delivery. So, I myself have to take rest along with focusing on newborn care. Even now it is quite difficult to walk or sit for me and perform my day-to-day activities. However, amongst all that I have a newborn to look after while handling my household chores.' - Female, 20s, rural*

*I always want somebody to visit and talk with me, hold the baby for some time, spend time with me. I crave getting support from my family members, especially my husband since she is his baby as well. I wish I could get some help during the night as I have not been able to get a good night's sleep since the last week. Me and my baby are alone most of the time. Sometimes, I think of attempting suicide by jumping from the terrace or running away somewhere far by leaving my baby. I have been constantly having multiple negative and suicidal thoughts and I feel low most of the times. And the worst part is that I have no one to share my thoughts with.' - Female, 30s, rural*

*I am not as happy as I was before when I had my first child. I feel tired, aggressive, and irritated without any reason. I don't feel like breastfeeding my baby or even holding the child and I want to create a space with my newborn. I feel like it's not my own baby due to which I have not been able to develop affection for him. AH these thoughts make me feel so guilty.' -Female, 30s, urban*

*'It is a difficult journey after childbirth. It is hard to deal with and handle the baby as a new mother. I have also experienced anger and extreme mood swings. It is too hard to balance work life with newborn care.' -Female, 20s, urban*

**Pregnancy and postpartum related taboos**

Many women face confusion about postpartum care, leading them to experiment with themselves and their child, potentially causing harm. Traditional superstitions, such as seeking treatment from healers like Jharfuke in Nepal, have deeply rooted in the minds of many new mothers. These beliefs, supported by respondents, are a result of societal pressures and blind superstitions.

*My child suffered from flu while traveling home and people here suggested me to visit Jharfuke (traditional healer) for the treatment. My sister-in-law told me about "Nazar" (evil eye) impact on newborn baby and new mothers which is why I put a black tika on my child before leaving the house.'-Female, 20s, rural*

*'Babies should wear kohl (black eyeliner) on their eyes to make their eyes big and beautiful and make their eyelashes longer. Oiling the baby out in the sun or beside the heat of the fire is required mandatorily.' -Female, 30s, urban*

*'We apply cow dung over the umbilical stump of a newborn to facilitate the healing process. It has been done by our ancestors over the years and we still continue doing so.'-Female, teenager, rural*

**Coping techniques or mechanisms**

Some mothers go through a tough time dealing with challenges on their own, without enough information, care, and support from their families. They start figuring things out for themselves, especially when things are difficult. They begin to engage in self-counselling and adapt to their situation. They focus on taking care of their child and work hard to become the best mothers they can be to their children.

*'During the challenging postpartum period, when family support was lacking, I found comfort at a local parlor. Sharing experiences with the employees made me realize that many others face similar phases during postpartum. They began spending more time with me, providing a supportive environment where I could express my thoughts and emotions freely.' -Female, 20s, urban*

*'Motherhood has definitely been a new challenge in my life at such a young age. Maybe with time, selfrealization and counselling will assist in changing mind set towards parenting and help embrace motherhood.' -Female, teenage, rural*

## DISCUSSION

In our study, the prevalence of postpartum depression was found to be 12.24% at a cut-off score of 12. When we compare these figures, our study's prevalence of 12.24% falls at the lower end of the spectrum. This is in contrast to the higher prevalence rates reported in many other studies conducted within the region, which show a range from 12.0% to 33.7%.^5-9,14,16-18^ The variation in these prevalence rates could be attributed to differences in cut-off value determining postpartum depression study methodologies, sample populations, length of post-partum period, or cultural factors influencing mental health reporting.

In the current study, the prevalence of postpartum depression has shown a marked improvement, decreasing from 14.7% in 2019^5^ to 12%. This follows a previous substantial rise from 4.9% in 2007^18^ to 30% in 2012^7^, with subsequent fluctuations in prevalence. Our findings suggest a comparatively lower incidence of postpartum depression, which may reflect specific regional or methodological differences but still warrants further investigation to understand the underlying factors contributing to these disparities.

The prevalence remained higher and continued to rise into 2020 in the study done outside the Kathmandu valley.^6,8,9,14,16^ The varying postpartum depression trends can be explained by better healthcare and awareness in Kathmandu Valley, leading to fluctuating rates, versus limited resources and higher stigma outside the valley, contributing to consistently high and rising rates. Socioeconomic factors and study differences also influence these trends. A metaregression analysis indicates that country development and income inequality significantly impact postpartum depression (PPD) rates. The findings show that PPD prevalence is higher in Southern and Western Asia compared to Northern America, Europe, and Oceania, highlighting the strong link between economic conditions and PPD epidemiology. ^4^

Current study shows a high rate of antenatal and postnatal checkups, with most women receiving satisfactory care from doctors and nurses. Intimate partner violence is minimal, and most pregnancies are planned. The low rates of gestational diabetes and pregnancy-induced high blood pressure in the study population reflect good prenatal care, contrasting with higher postpartum depression rates seen in other regions with less access to such care.^4^ Similarly, the study's low postpartum depression rate may be influenced by the high percentage of planned pregnancies, minimal vaginal tearing, and flexible gender preferences. These factors generally contribute to reduced stress and better mental health, potentially lowering the risk of postpartum depression which is supported by number of studies.^19-23^

The qualitative findings of the current study showed that the mothers have failed to receive the required support in handling the newborn child as they reported receiving compromised social, family and husband support during their postpartum period which is supported by a similar study done in Korea.^24^ Various similar studies also agreed on the strong need of social support to manage the physical and mental challenges that mothers faced through the postpartum period.^24-26^ Insomnia, daytime being alone, conventional family structures, child health, and work-life balance are some of the factors that affect postpartum depression in mothers and can have a negative impact on their general well-being.^24,27^ The findings related to taboo further illustrates how cultural practices and beliefs around newborn care can intersect with maternal experiences. The pressure to conform to traditional practices, combined with concerns about the baby's health and wellbeing, can contribute to maternal stress and potentially affect mental health, including the risk of postpartum depression which is supported by an exploratory mixed method study on postpartum depression.^28,29^

Various factors such as patient's mode of delivery, pregnancy/delivery complication and afterbirth pain could have played vital role in their occurrence of depressive symptoms which is supported by the series of similar previous studies in the global context.^17,18,25,30^ Previous study agreed on providing proper information on safe motherhood and newborn health care knowledge^18^ since poor knowledge about pregnancy and postpartum changes can lead mothers to rely on social media, friends, or traditional methods, causing confusion and superstitions.^28^ This can result in adverse health outcomes for both the mother and child. However, the qualitative findings of the current study reveal that external support and shared experiences can significantly reduce feelings of isolation and offer emotional relief to mothers. Studies emphasize that self-awareness and professional counselling are crucial for managing the emotional difficulties of early motherhood.^31,32^ Promoting education and awareness about both physical and mental health is crucial, as it allows for the customizing of counselling strategies to individual coping mechanisms, thereby enhancing their effectiveness and ensuring that new mothers receive the comprehensive support they need to navigate the complexities of postpartum depression.

## CONCLUSION

Postpartum depression prevalence was lower than similar settings. Coping mechanisms included sharing, self-counselling, and self-realization. Comprehensive perinatal care assessments are crucial for managing morbidities. Timely depression screening in postpartum mothers is essential for mitigating risks and adverse outcomes.
